# The effect of parental presence on the anxiety during first dental treatment in children

**DOI:** 10.1080/00016357.2023.2262019

**Published:** 2024-03-26

**Authors:** Serhat Karaca, Belen Şirinoğlu Capan

**Affiliations:** a Faculty of Dentistry, Department of Pediatric Dentistry, Erzincan Binali Yıldırım University, Erzincan, Turkey; b Faculty of Dentistry, Department of Pediatric Dentistry, Istanbul University-Cerrahpasa, Istanbul, Turkey

**Keywords:** Dental anxiety, children, parental presence, heart rate

## Abstract

**Objective:**

The aim of this study was to evaluate the effect of parental presence on dental anxiety in children during dental treatments.

**Materials and methods:**

The study was conducted with 194 children between January-April, 2020. The children were randomly divided into two subgroups. Children in group-I were treated in parent’s presence (pp), and in group-II in parent’s absence (pa). The Wong-Baker FACES Pain Rating Scale (WBFPS) and The Modified Dental Anxiety Scale (MDAS) were used for subjective measurements, whereas the objective measurement was performed by measuring the heart rate.

**Results:**

The mean age of 194 children was 6.26 ± 1.15years, ranging from 5-8years of age. The mean MDAS score of all children was 15.1 ± 4.72. No significant correlations were found in terms of dental anxiety between the children’s gender and age with heart rate, WBFPS and MDAS scores. Preoperative WBFPS scores (6.83 ± 1.04 pp and 7.01 ± 0.93pa) were higher than postoperative scores (5.34 ± 2.11 pp and 5.74 ± 2.04pa), with no statistically significant difference. Although there was no statistically significant results, the paediatric dentist observed a deterioration in the children’s behavior throughout the sessions in group-II compared to children in group-I.

**Conclusions:**

Parental presence has no statistically significant effect on dental anxiety in children during dental treatments.

## Introduction

Dental anxiety is defined as a state of intense restlessness and delusion that cannot be fully expressed due to fear of dental treatment [1]. Anxiety can be seen in any age, gender and social class, but educational status, personality traits, gender, age, and past dental experiences affect patients’ anxiety levels [2,3].

As the fear of dental treatments constitutes to be a major problem in oral and dental health around the world, it is important to prevent this fear, especially in terms of improving the quality of life of children [4,5]. Many dentists think that dental anxiety affects the relationship between patient and physician, causing an inadequate diagnosis and treatment planning that results in poor oral health [6,7]. Dahlander et al. [8] stated that, as a result of children not being able to overcome their fear of dentists, they may transfer their fears to their own children when they become parents themselves.

In order to determine the fear and anxiety of the paediatric patients many techniques are used such as scoring of behaviors, psychometric measures, psychological tests and projective techniques. There are many different scales for measuring dental anxiety levels. Humphris et al. [9] created the Modified Dental Anxiety Scale (MDAS) in 1995. MDAS is specific for dental anxiety and is advantageous because it is economical in short and population-based research [10]. In psychological techniques, it is aimed to obtain information about the severity of fear and anxiety by making measurements such as blood pressure, pulse and oxygen saturation level by using special equipments [11,12].

Children’s responses in the presence of parents in the treatment environment can have different results, from very useful to extremely challenging [13]. The purpose of determining the effect of parental presence or absence is to improve the child’s cooperation, to prevent negative or avoidant behaviors, to establish proper dentist patient roles, to strengthen effective communication between the dentist, the child and the parents, to reduce anxiety and to achieve a positive treatment experience [14,15].

Behavior management techniques in paediatric dentistry aim to reduce the fear and anxiety of the child, to provide permanent behavior change in the child and to provide quality dentistry services [16,17]. With the relationship between the dentist and the child, the fear and anxiety the child experiences can be reduced, the child become self-confident, so the child can feel more relaxed during dental treatments [18].

The aim of this study was to evaluate the effect of parental presence on dental anxiety in their children during dental treatments.

## Materials and methods

### Study group

Over a 3 months period 194 children aged between 5-8 years old attended to the peadiatric dental clinic of Erzincan Binali Yıldırım University in Turkey (January 2020- April 2020). As the inclusion criteria: children were classified in group 1 or 2 on the Frankl scale, presenting for a restorative treatment in the primary first molars, presenting for dental treatment without pulp involvement or pain, and both the children and parents could speak Turkish. Furthermore children with an anormal developmental level (requiring special education) or a mental illness, children with a past experience of surgery or those with chronic illnesses requiring repeated hospitalization were excluded from the study. All the parents were informed and asked to participate in the study. This study was approved by the Erzincan Binali Yildirim University Ethics Committee with 05/09 numbered decision on 29/04/2020.

### Procedure

All the children were ramdomly divided into two groups. In the first group the parents were present in the operating room, whereas in the second group, the parents remanied in the waiting room. All treatment sessions were held in the morning. Behavior management techniques, such as Tell–Show-Do, voice control, modeling and contingency management, were used during dental treatments for all the children. Only one peadiatric dentist participated in the study. Before and after each treatment session, the dentist, child and parent were asked to report on the child’s anxiety.

### Subjective measurement

In each session the subjective measurement of dental anxiety was done in the waiting room. While different scales are used to evaluate dental anxiety levels, in this study, the Modified Dental Anxiety Scale (MDAS) form was used to determine the level of dental anxiety. This form, which consists of a total of 5 questions, has an additional question related to injection to the ‘Corah’s Dental Anxiety Scale’ to determine the level of anxiety and developed by Humphris et al. [9] For each of the answer options, a scoring method between 1 and 5 points is used. The lowest total score on the whole questionnaire is 5 and the highest score is 25. The scale studies conducted in Turkey was reported to be valid and reliable [10]. The limit of dental anxiety for MDAS was accepted as ≥ 15, as described in Tunç et al.’s study [10]. The Wong-Baker FACES Pain Rating Scale (WBFPS) was applied to determine the level of dental anxiety of the children before and after the sessions [19]. WBFPS contains a row of six faces ranging from smiling to crying in combination with a visual scale of 0-10. For a proper calibration, all fear measurements were performed by a single examiner.

### Objective measurement

The objective measurement of dental anxiety of the children was done by measuring the heart rate using a portable pulse oximeter. The heart rate scores were monitored and recorded in every 5 min by using a portable pulse oximeter for approximately 20 min of dental treatment.

### Statistical analysis

All data analysis was performed using the Statistical Package for the Social Sciences (SPSS) version 22 data processing software (IBM corp, Armonk NY, USA). To exclude all differences between the groups the independent samples T test was used, whereas for differences in the behavior and anxiety scores between the groups, the Mann Whitney U test was used. The level for statistical significance was set at *p*<0.05.

## Results

Out of the 194 children, 102 were girls (mean age 6.4 ± 1.1 years) and 92 were boys (mean age 6.1 ± 1.2 years). The mean age of the children was 6.26 ± 1.147 years, ranging from 5-8 years of age. There were no statistically significant differences between children’s age and genders with dental anxiety regardless of whether the parents were present or absent. No significant correlations were noted between the children’s gender and age with heart rate, the WBFPS and MDAS scores.

Table 1 shows the means and standard deviations of the variables according to the absence or the presence of the parents in this study. There were no statistically significant differences for any of the variables regarding the parents’ presence or absence. It was observed that there was a decrease in the children’s discomfort when a parent was present in the operatory room. A deterioration in the child’s behavior during the sessions was observed by the paediatric dentist in children whose parents were outside the operatory compared to those whose parents were present, but there was no statistically significant difference in the data obtained between the two groups (parents absent and parents present).

The mean MDAS scores of the total sample of 194 children was 15.1 ± 4.72. Of those children, 102 were treated with parents present in the operatory room and the other 92 children’s parents were in the waiting room. Table 1 showed that there were no statistically significant correlations in MDAS scores in the groups of parents who were present or absent (Table 1).

The WBFPS was used for determining the dental anxiety of the children before and after the treatments. Preoperative WBFPS scores were higher than postoperative scores in two groups with a statistically insignificant difference. No significant correlations were found in the preoperative and postoperative WBFPS ratings between the parents present and parents absent groups (Table 1).

**Table 1:** Mean scores and standard deviations of the variables regarding to the presence or absence of the parents.

	Parent present	Parent absent	p value
	Mean ± SD	Mean ± SD	
MDAS	14.6 ± 4.69	15.6 ± 4.75	0.45
Wong-baker FACES pain	6.83 ± 1.04	7.01 ± 0.93	0.18
rating scale preoperative scores			
Wong-baker FACES pain	5.34 ± 2.11	5.74 ± 2.04	0.15
rating scale postoperative scores			
Heart rate in 5 min	90 ± 4.59	90.3 ± 4.44	0.79
Heart rate in 10 min	94.6 ± 4.58	95.2 ± 3.90	0.39
Heart rate in 15 min	99.3 ± 4.97	99.5 ± 4.44	0.76
Heart rate in 20 min	97.8 ± 5.54	98.1 ± 4.63	0.51

The heart rate levels were detected at different times during the treatments and compared with the Mann–Whitney U test. Table 1 shows the mean and standard deviation (SD) for the heart rate scores for each stage in both groups. As can be seen, the heart rate scores show no significant differences (Table 1).

## Discussion

Parents play an important role in the cooperation of paediatric patients. There are different opinions about whether the child should given treatment with or without their parents’ presence. Kostanos et al. [20] have suggested that the presence or absence of parents can be a powerful behavior management tool. Dentists who investigating children’s behavior; observed that children develop their behavior when the parents are not with them in the operating room. Nevertheless, there is no consensus regarding the age at which parents should be present with the child during dental treatments.

There are many studies that have argued that the absence of the parents from the operating room can improve the behavior of paediatric patients [6,12]. On the other hand, some authors have pointed to benefits of the presence of the parents in the operatory for reducing the dental anxiety children [21,22]. The aim of this study was to subjectively and objectively evaluate the impact of the presence or absence of the parents with regard to the anxiety of children.

There was no significant difference between genders and ages with dental anxiety in this study. The same pattern of anxiety was obtained in males and females and also in all different age groups in the treatment sessions. These outcomes are consistent with the findings of previous studies in the literature. [3,6,13].

In many studies, heart rate is used as an objective indicators of physiological fear responses [11,12]. The measurement of heart rate by using a portable pulse oximetery was preferred in this study as he is a reliable and sensitive indicator of dental anxiety. Mendoza-Mendoza et al. [19] carried out a study involving 303 children between 3 and 12 years of age by using WBFPS with the aim of evaluating the correlation between dental anxiety and previous negative dental experiences. Guinot Jimeno et al. [23] and Vasiliki et al. [24] used WBFPS in their research for measuring dental anxiety of the participants. We also used WBFPS to assess the changes in the dental anxiety of the children in our study. Many scales have been developed to evaluate dental anxiety. As in many studies, MDAS was preferred in this study because it is an easy and reliable method [24,25].

The findings of this study indicated that there was no statistically significant correlations between the anxiety of children during dental treatment and whether the parent was present or absent. However, according to the subjective datas there was an insignificant difference in the children’s attitudes and responses. This results are in agreement with a similar previous study [26]. In our study, we observed that there were no significant differences in MDAS, WBFPS and heart rate data whether the parents were present or absent in the operatory.

According to the literature reviews, studies conducted in recent years have reported that parental presence/absence in the operatory room has no effect on the anxiety level of children [24,26,27]. Similarly, Shindova and Belcheva [28] and Acharya et al. [29] observed in their studies that parental presence has no effect on dental anxiety in children at aged 6-12 in clinical examination. These results are consistent with our results. The fact that a systemic review published in 2021 reported that the parental presence in the operatory room did not affect the behavior, fear and anxiety of children during dental treatment supports our findings [15]. In contrast, some studies in the literature have mentioned that parental presence reduces the level of anxiety in children during dental treatments [13,30,31].

Behavior management techniques aim to alleviate the anxiety in the child and to promote the child’s positive attitude toward oral health care. It is recommended that the child is fully evaluated and that different methods are applied in different processes customized for each child [13].

In conclusion, parental presence has no statistically significant effect on dental anxiety in children during dental treatments. However, it was found that children seem to be more positive in the presence of their parents in the dental operatory.

## Data Availability

The data that support the findings of this study are available from the corresponding author upon reasonable request.
